# Robotic techniques benefit anaesthesia too

**DOI:** 10.1177/01410768231160278

**Published:** 2023-03-09

**Authors:** Sean P Hughes, Iain Macintyre

**Affiliations:** Sean P Hughes: Faculty of Medicine, Imperial College London, Hammersmith Hospital, London, SW7 2AZ, UK; Iain Macintyre: Royal College of Surgeons of Edinburgh, Edinburgh EH8 9DW, UK.

Dear Sir,

We thank Charles Pither for his interest in our paper. His suggestion that we ‘forgot or downplayed the role of the anaesthetist’ is not correct.^1^ In our article, we emphasised the remarkable improvements in surgery brought about by anaesthesia when we wrote ‘The introduction of anaesthesia was clearly ground-breaking’. William Osler, later wrote, ‘Search the scriptures of human achievement and you cannot find any to equal the beneficence of the introduction of Anaesthesia, Sanitation, with all that includes, and Asepsis’.^1^

The point we were making in our paper was that the fear of inflicting pain did not stop surgeons from carrying out painful procedures in pre-anaesthetic days. Procedures including trepan, limb amputation, aneurism ligation, hernia repair, mastectomy and lithotomy were all in the surgeon's repertoire even before anaesthesia was introduced. Even ovarian cystectomy was performed in the pre-anaesthesia period. Horrendous though it was, pain per se did not kill patients, but postoperative sepsis did, and it was mortality from sepsis that deterred surgeons from operating.

Charles Pither goes on to suggest that anaesthetists will always be required, implying that they will never be replaced by artificial intelligence or robotic systems. It seems, however, that anaesthesia, like surgery, will inevitably benefit from these technological advances. Hashimoto et al.^2^ write ‘anaesthesiology as a field is well positioned to potentially benefit from advances in artificial intelligence as it touches on multiple elements of clinical care’. They also recommend that anaesthetists ‘should partner with other providers (surgeons, interventionalists intensivists, nurses) and patients to help develop the strategy for the optimal use of artificial intelligence’. Furthermore, Zaouter et al.^3^ evaluated the use of pharmacological, mechanical and cognitive robots in anaesthesia, clearly advocating a role for robots of different sizes and structures in anaesthesia.

Nowhere did we predict, as Charles Pither suggests, that the surgeon would be ‘replaced by a robot’. While surgeons and anaesthetists will increasingly adopt innovative technologies to improve patient safety and outcomes, we believe that for the foreseeable future, surgeons and anaesthetists, not robots, will remain in overall control of operative procedures.

## Declarations

### ORCID iD

Sean P Hughes https://orcid.org/0000-0002-4710-534X
